# Supplementary material for: Integration of Infinium and Axiom SNP array data in the outcrossing species Malus × domestica and causes for seemingly incompatible calls
Source: BMC Genomics. 2021 Apr 7;22:246. doi: 10.1186/s12864-021-07565-7 (PMC8028180; doi:10.1186/s12864-021-07565-7)
Supplement: Supplementary file 4 — Additional file 4. Cluster plot examples for classifications of compatible SNPs from Table 3. [file 12864_2021_7565_MOESM4_ESM.pptx]

## Slide 1
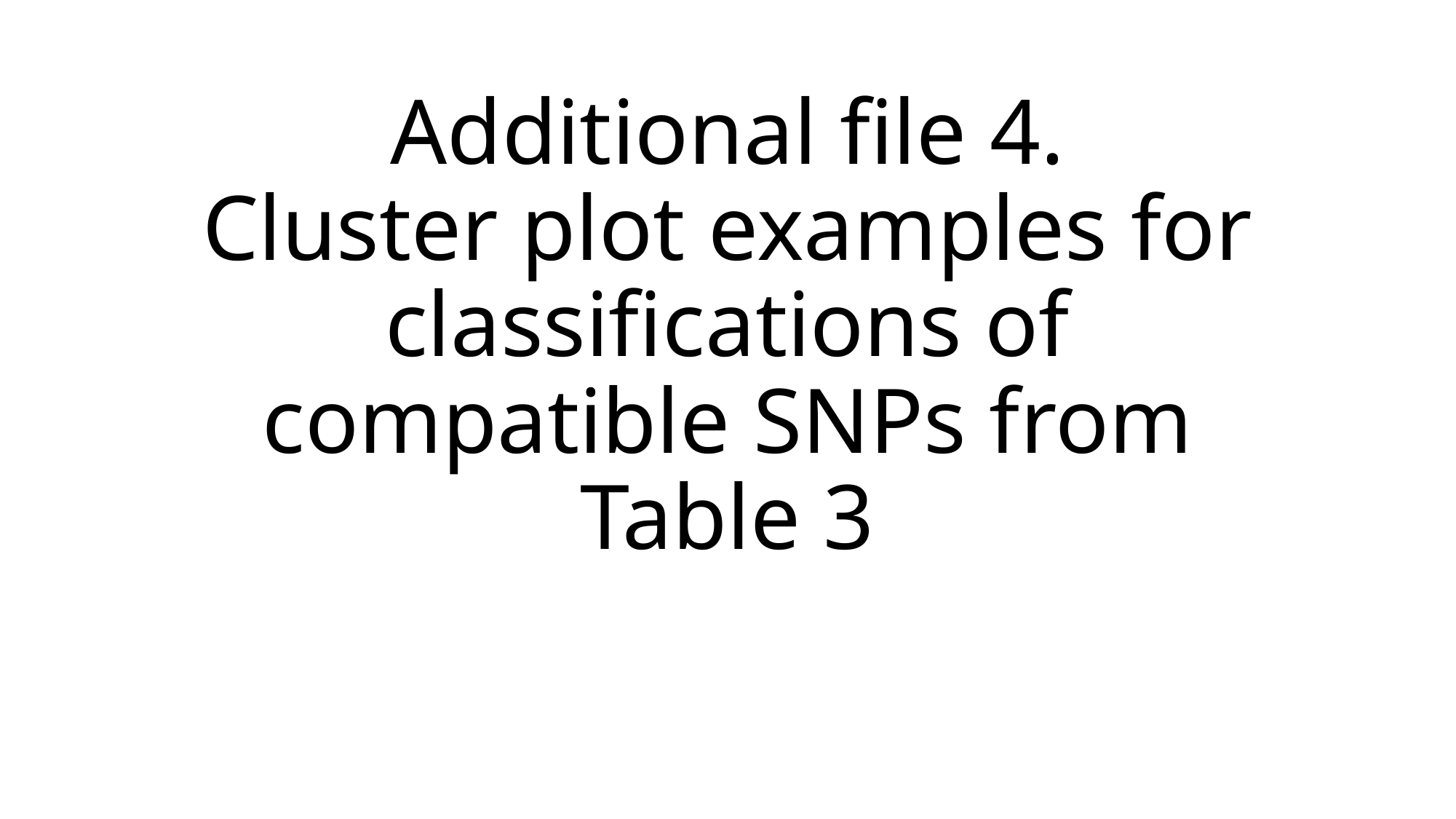

# Additional file 4. Cluster plot examples for classifications of compatible SNPs from Table 3

## Slide 2
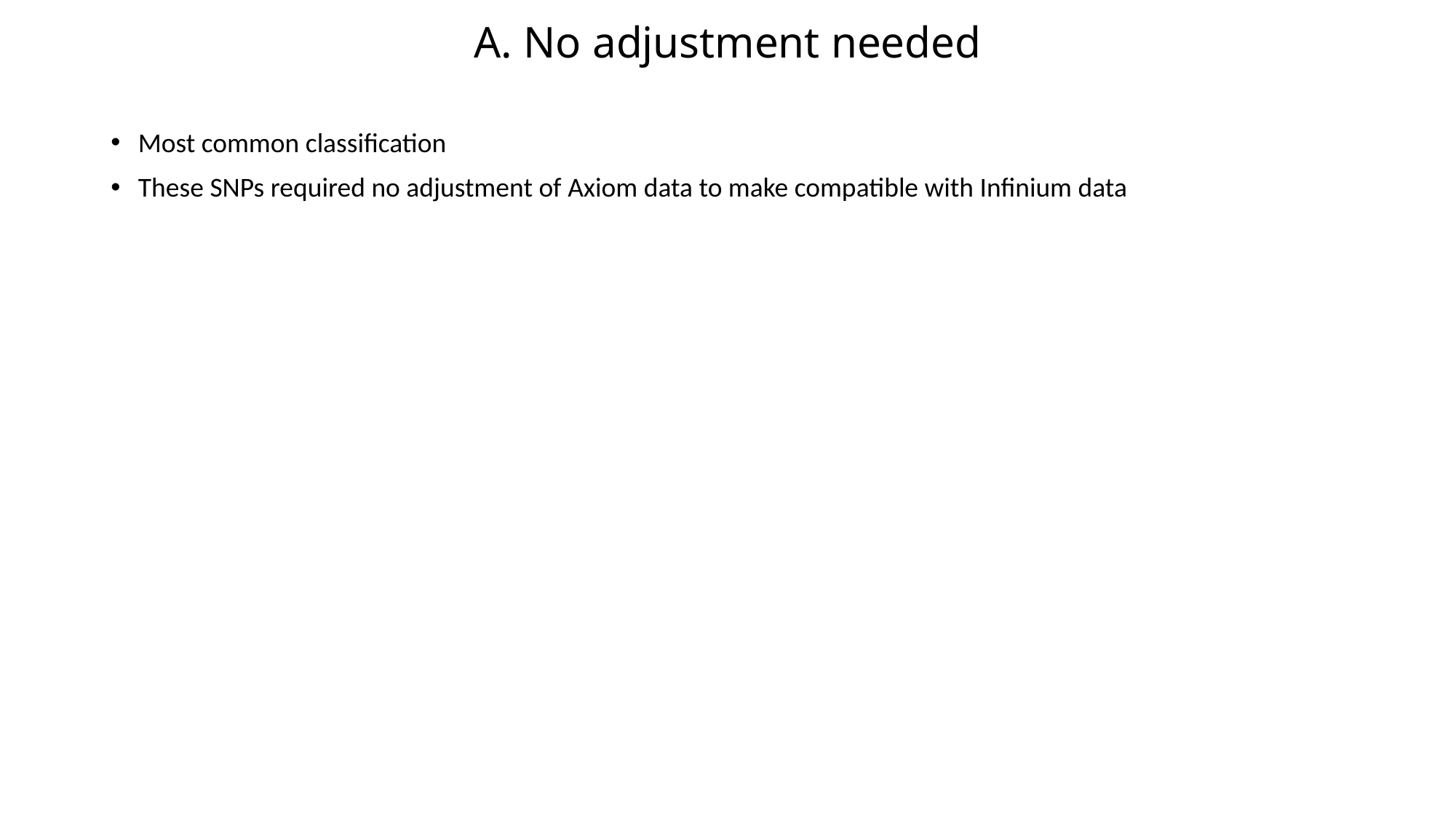

# A. No adjustment needed
Most common classification
These SNPs required no adjustment of Axiom data to make compatible with Infinium data

## Slide 3
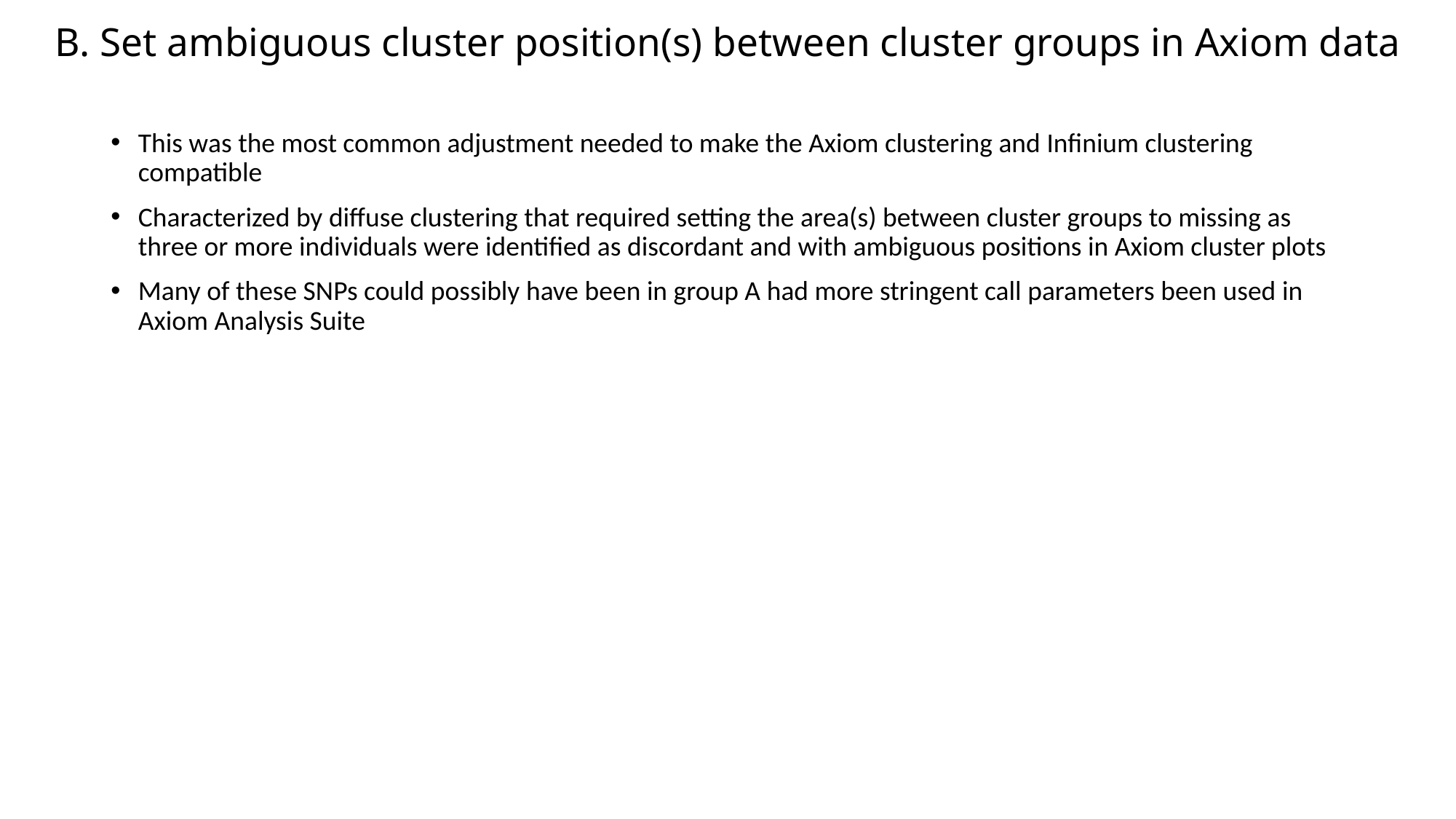

# B. Set ambiguous cluster position(s) between cluster groups in Axiom data
This was the most common adjustment needed to make the Axiom clustering and Infinium clustering compatible
Characterized by diffuse clustering that required setting the area(s) between cluster groups to missing as three or more individuals were identified as discordant and with ambiguous positions in Axiom cluster plots
Many of these SNPs could possibly have been in group A had more stringent call parameters been used in Axiom Analysis Suite

## Slide 4
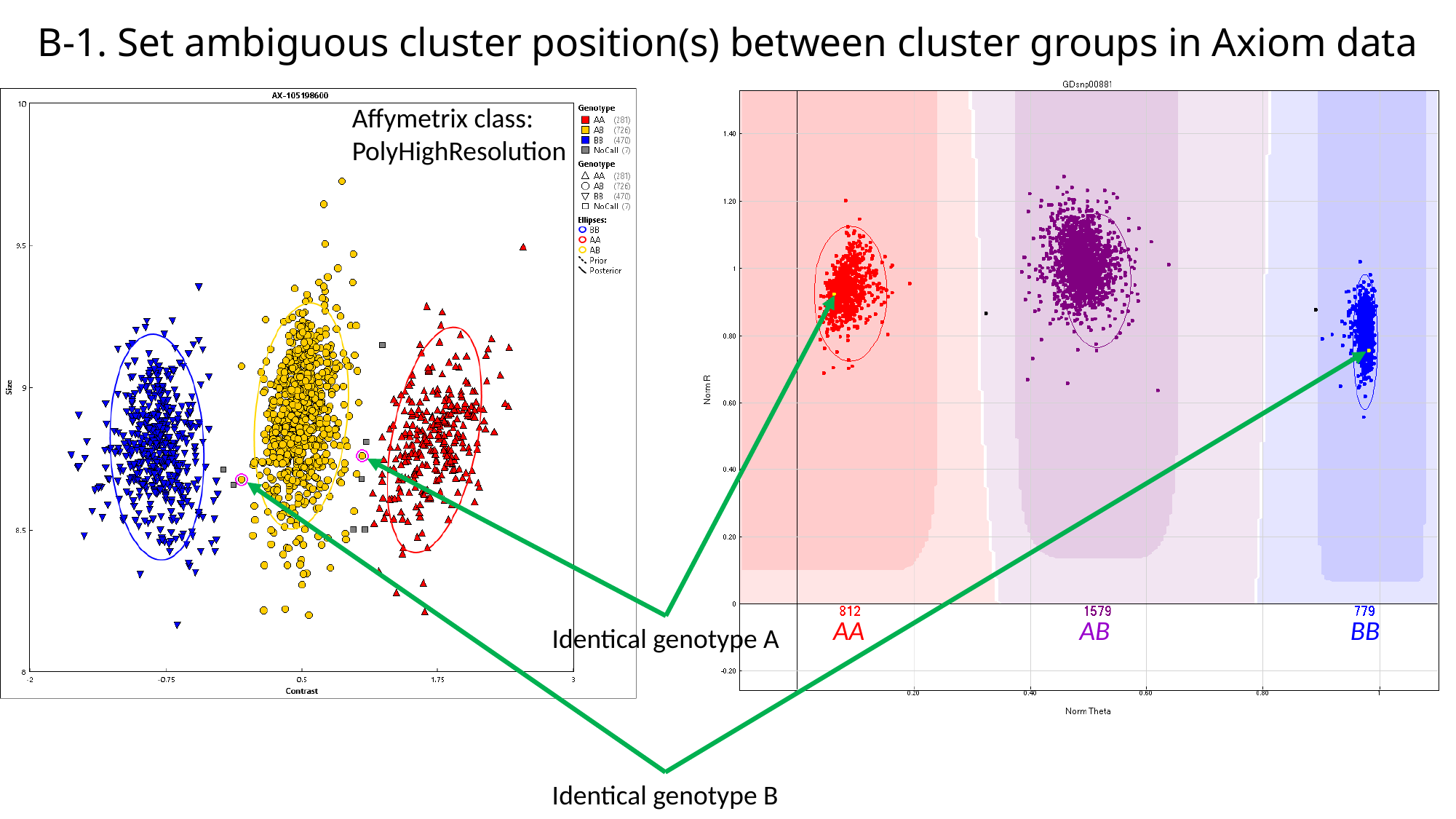

# B-1. Set ambiguous cluster position(s) between cluster groups in Axiom data
Affymetrix class:
PolyHighResolution
AA
AB
BB
Identical genotype A
Identical genotype B

## Slide 5
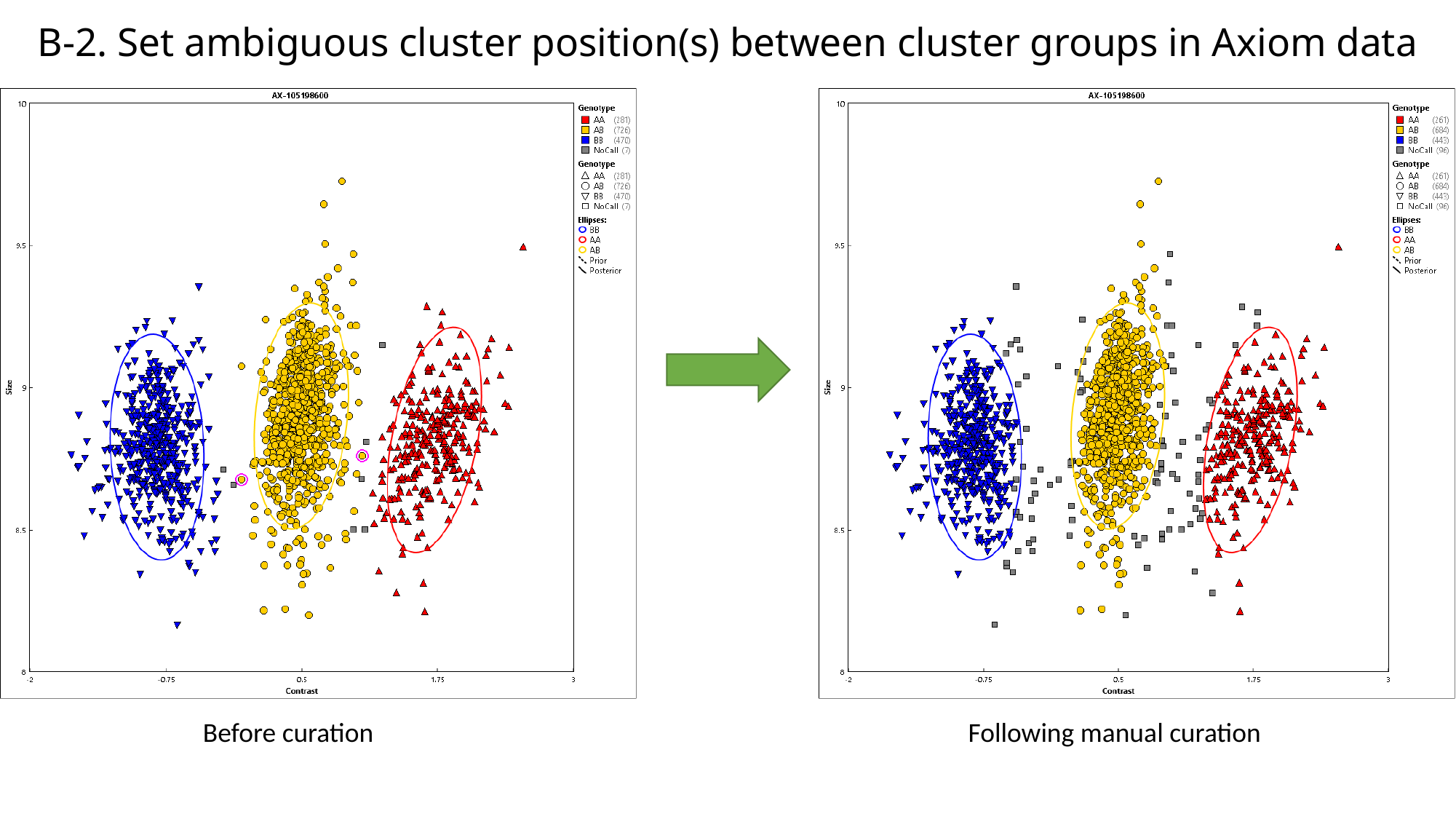

# B-2. Set ambiguous cluster position(s) between cluster groups in Axiom data
Before curation
Following manual curation

## Slide 6
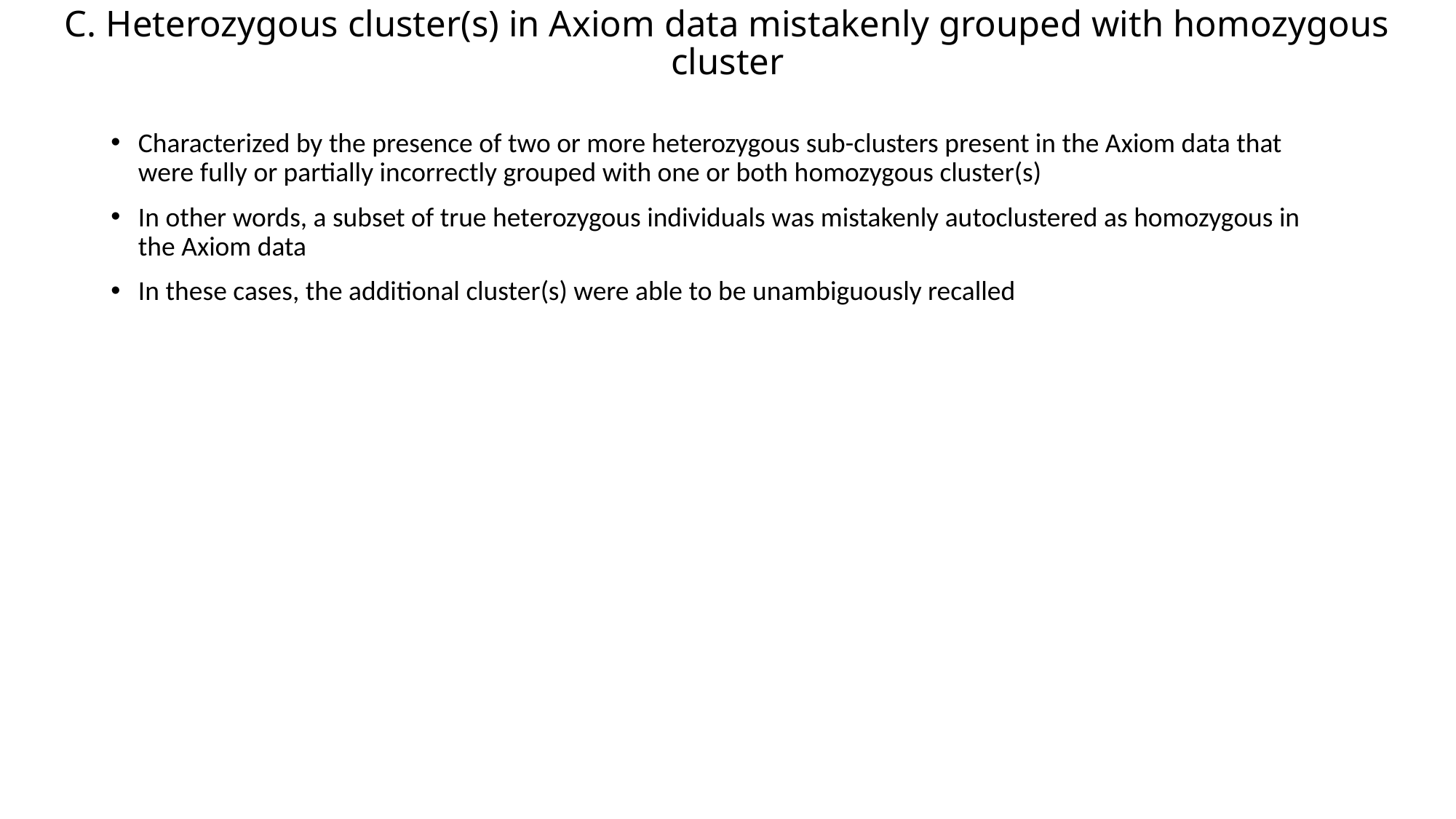

# C. Heterozygous cluster(s) in Axiom data mistakenly grouped with homozygous cluster
Characterized by the presence of two or more heterozygous sub-clusters present in the Axiom data that were fully or partially incorrectly grouped with one or both homozygous cluster(s)
In other words, a subset of true heterozygous individuals was mistakenly autoclustered as homozygous in the Axiom data
In these cases, the additional cluster(s) were able to be unambiguously recalled

## Slide 7
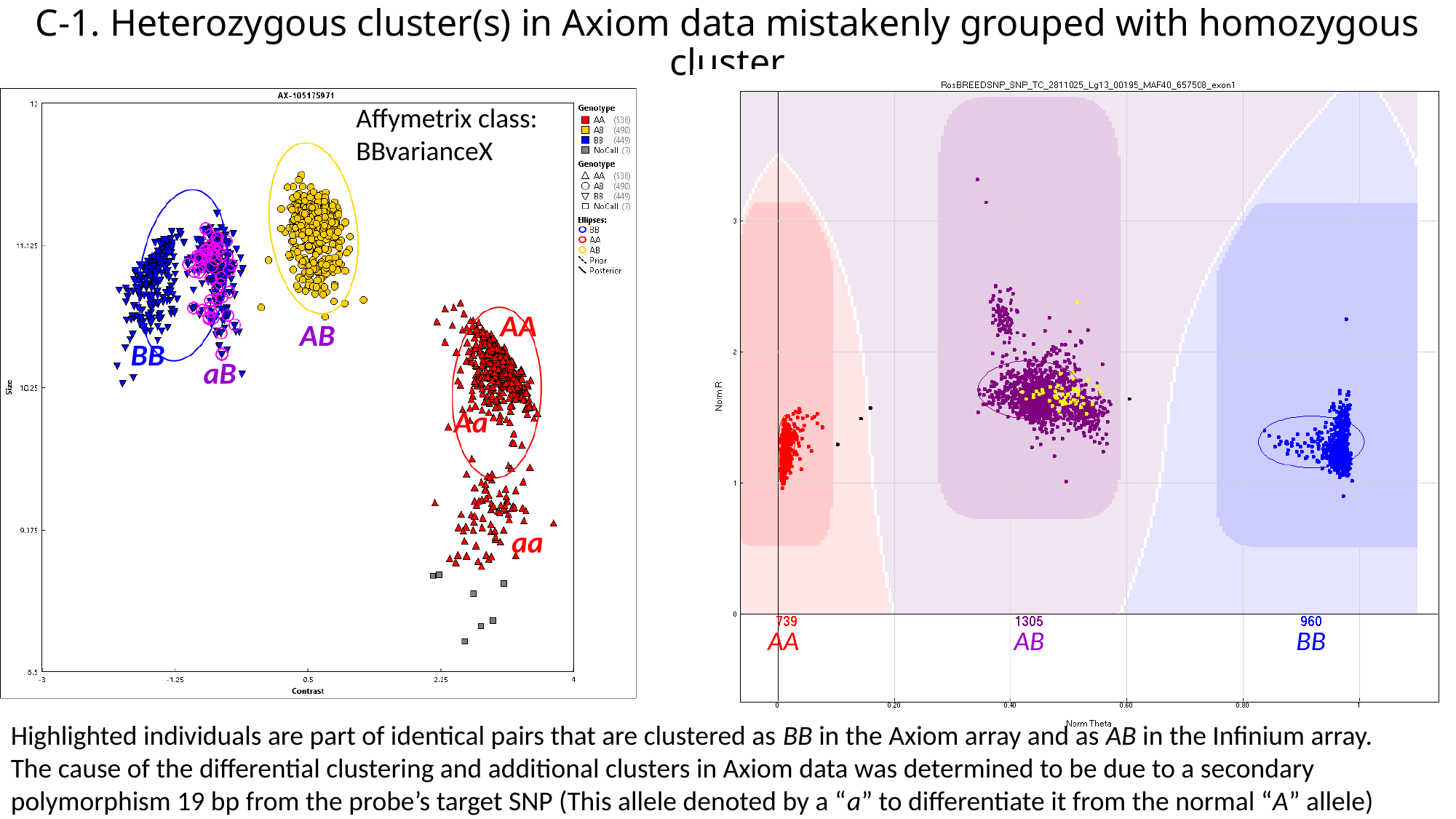

# C-1. Heterozygous cluster(s) in Axiom data mistakenly grouped with homozygous cluster
Affymetrix class:
BBvarianceX
AA
AB
BB
aB
Aa
aa
AA
AB
BB
Highlighted individuals are part of identical pairs that are clustered as BB in the Axiom array and as AB in the Infinium array. The cause of the differential clustering and additional clusters in Axiom data was determined to be due to a secondary polymorphism 19 bp from the probe’s target SNP (This allele denoted by a “a” to differentiate it from the normal “A” allele)

## Slide 8
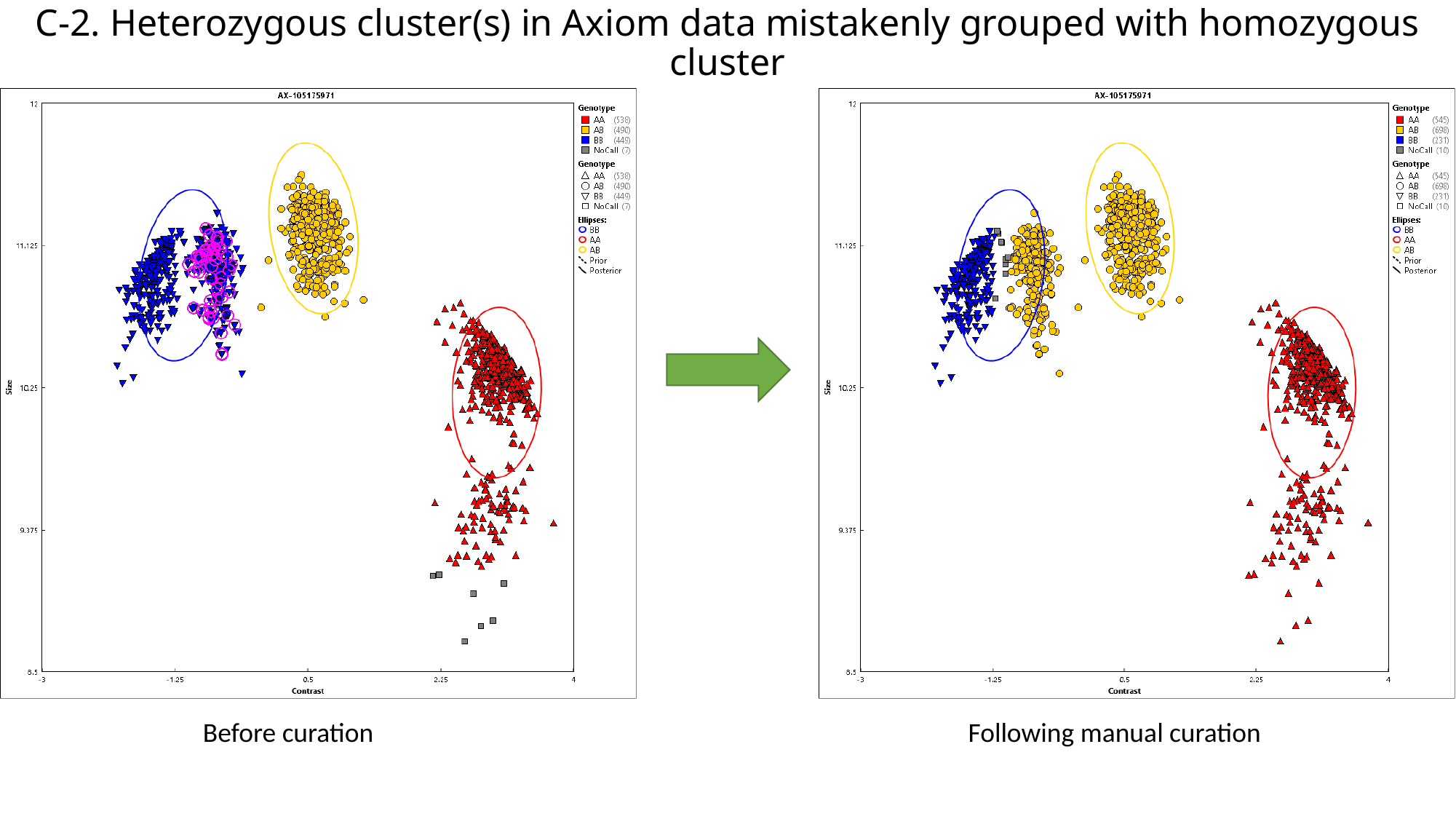

# C-2. Heterozygous cluster(s) in Axiom data mistakenly grouped with homozygous cluster
Before curation
Following manual curation

## Slide 9
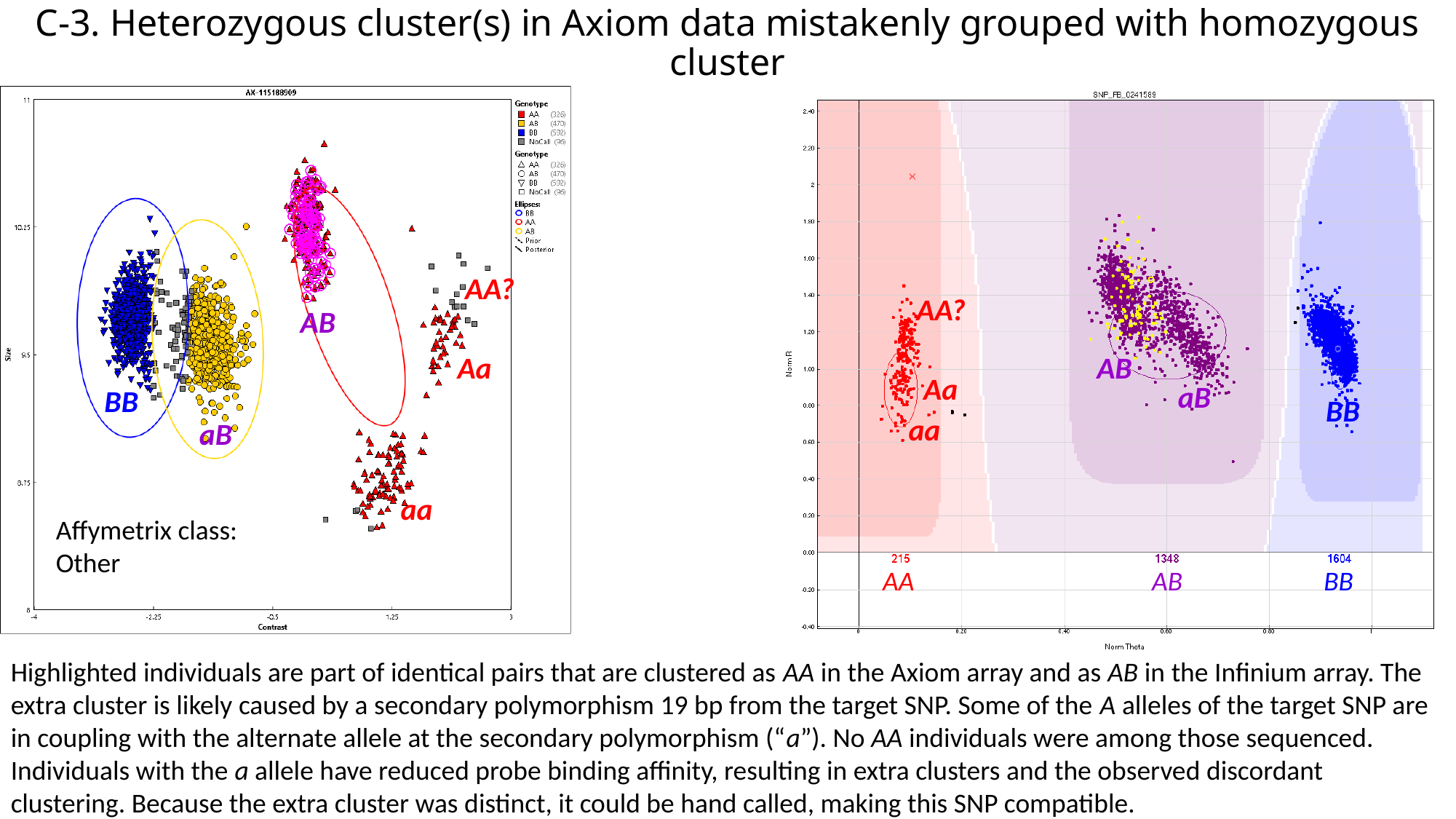

# C-3. Heterozygous cluster(s) in Axiom data mistakenly grouped with homozygous cluster
AA?
AA?
AB
Aa
AB
Aa
aB
BB
BB
aa
aB
aa
Affymetrix class:
Other
AA
AB
BB
Highlighted individuals are part of identical pairs that are clustered as AA in the Axiom array and as AB in the Infinium array. The extra cluster is likely caused by a secondary polymorphism 19 bp from the target SNP. Some of the A alleles of the target SNP are in coupling with the alternate allele at the secondary polymorphism (“a”). No AA individuals were among those sequenced. Individuals with the a allele have reduced probe binding affinity, resulting in extra clusters and the observed discordant clustering. Because the extra cluster was distinct, it could be hand called, making this SNP compatible.

## Slide 10
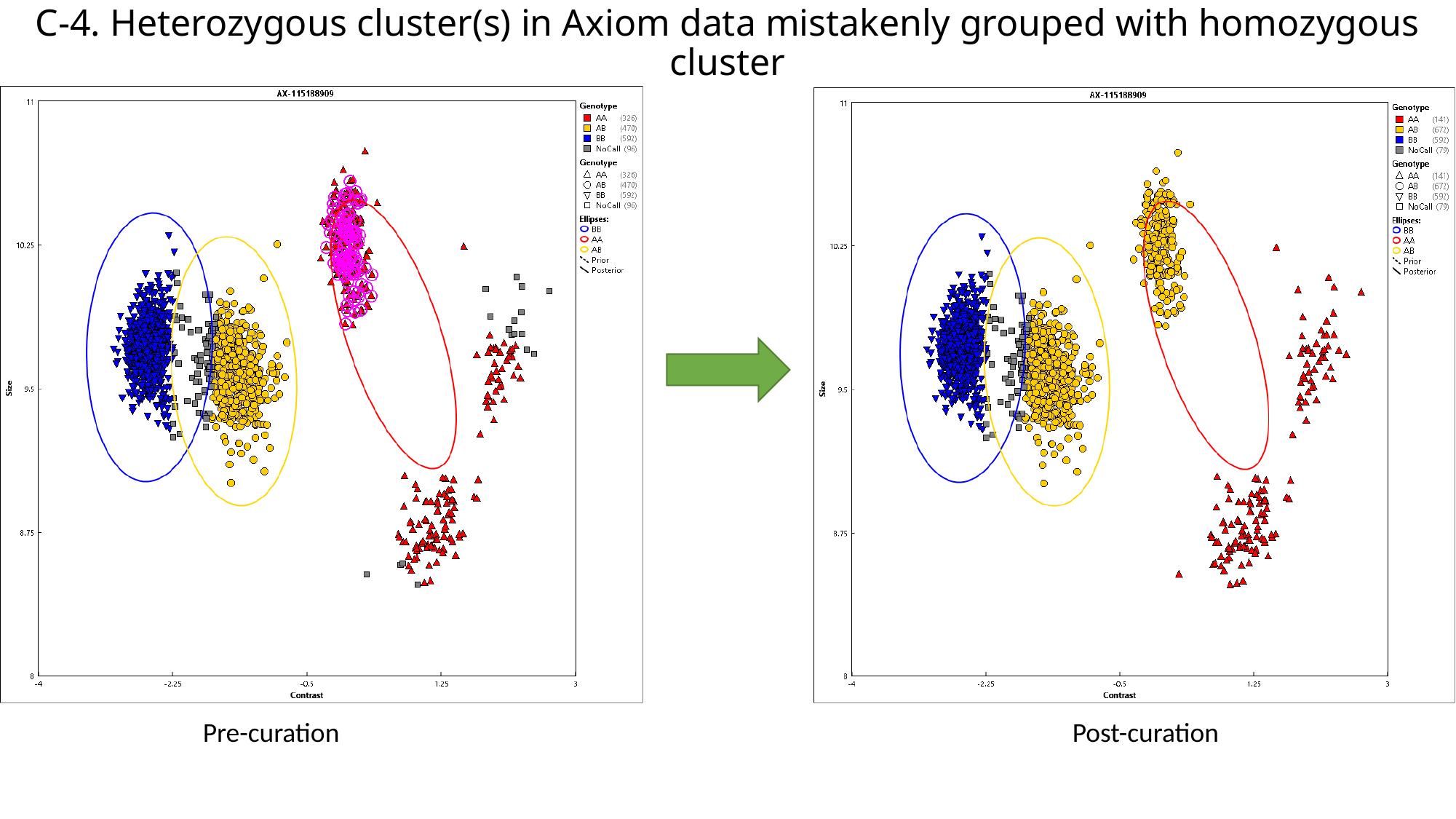

# C-4. Heterozygous cluster(s) in Axiom data mistakenly grouped with homozygous cluster
Pre-curation
Post-curation

## Slide 11
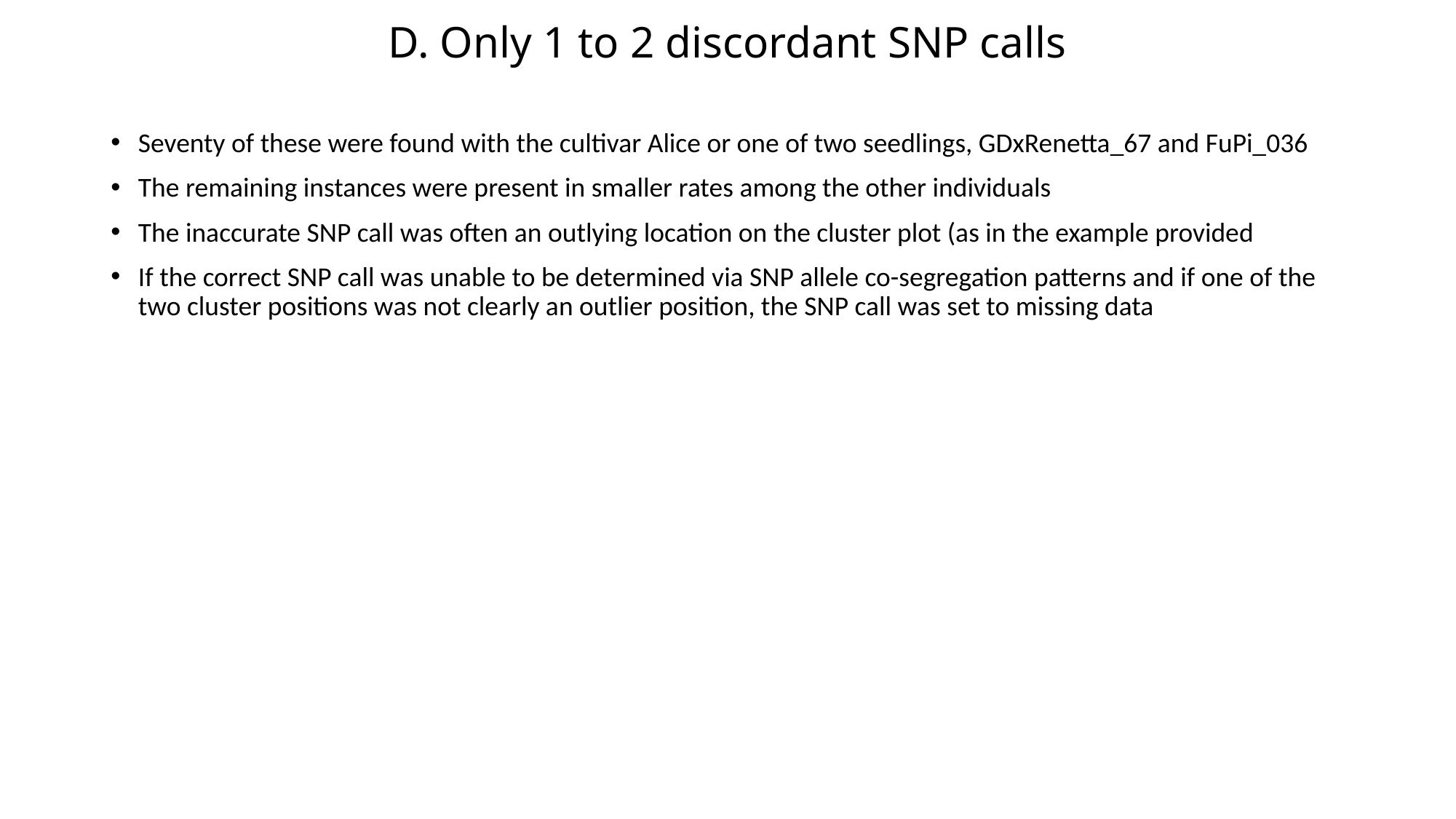

# D. Only 1 to 2 discordant SNP calls
Seventy of these were found with the cultivar Alice or one of two seedlings, GDxRenetta_67 and FuPi_036
The remaining instances were present in smaller rates among the other individuals
The inaccurate SNP call was often an outlying location on the cluster plot (as in the example provided
If the correct SNP call was unable to be determined via SNP allele co-segregation patterns and if one of the two cluster positions was not clearly an outlier position, the SNP call was set to missing data

## Slide 12
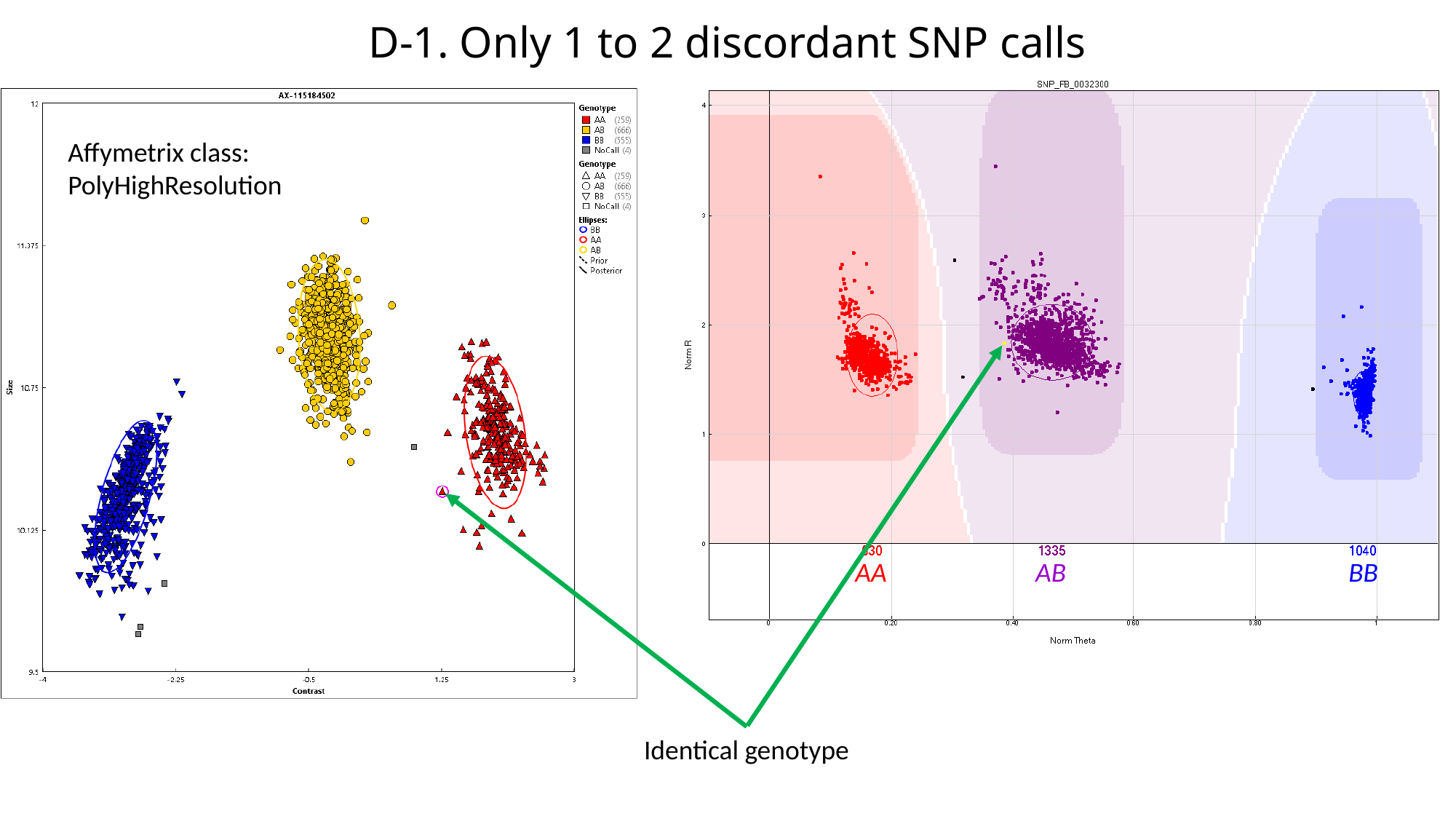

# D-1. Only 1 to 2 discordant SNP calls
Affymetrix class:
PolyHighResolution
AA
AB
BB
Identical genotype

## Slide 13
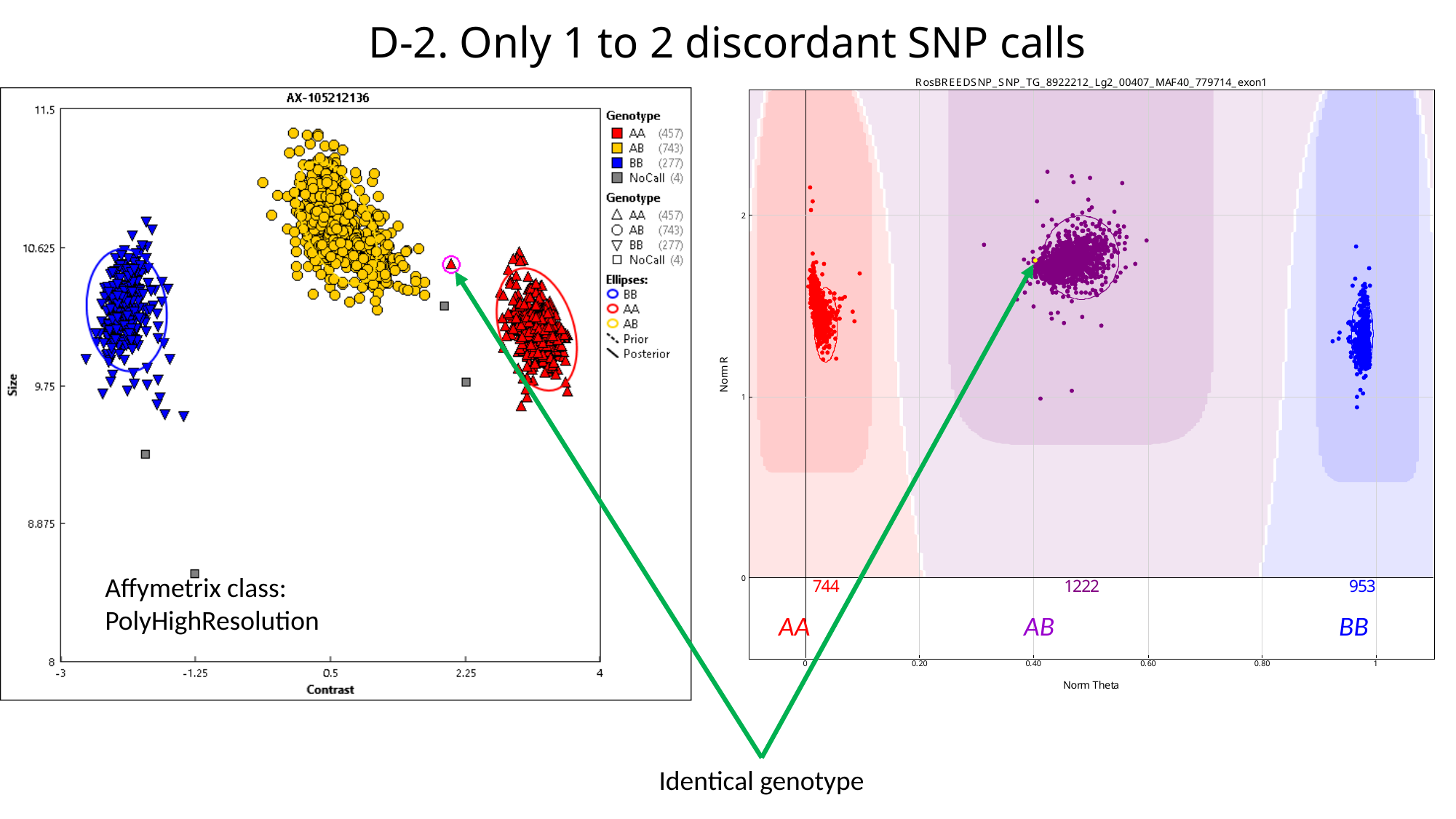

# D-2. Only 1 to 2 discordant SNP calls
Affymetrix class:
PolyHighResolution
AA
AB
BB
Identical genotype

## Slide 14
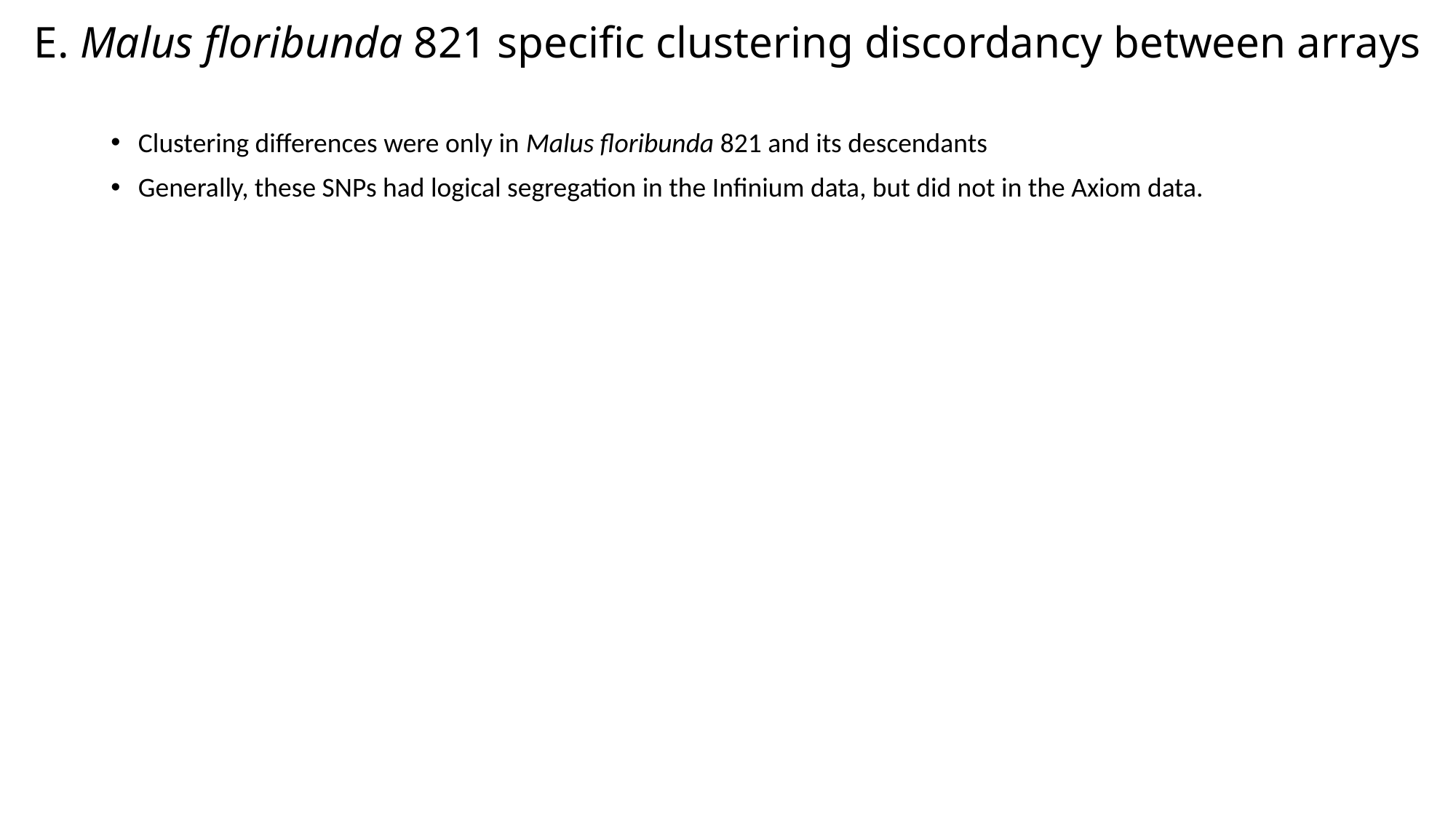

# E. Malus floribunda 821 specific clustering discordancy between arrays
Clustering differences were only in Malus floribunda 821 and its descendants
Generally, these SNPs had logical segregation in the Infinium data, but did not in the Axiom data.

## Slide 15
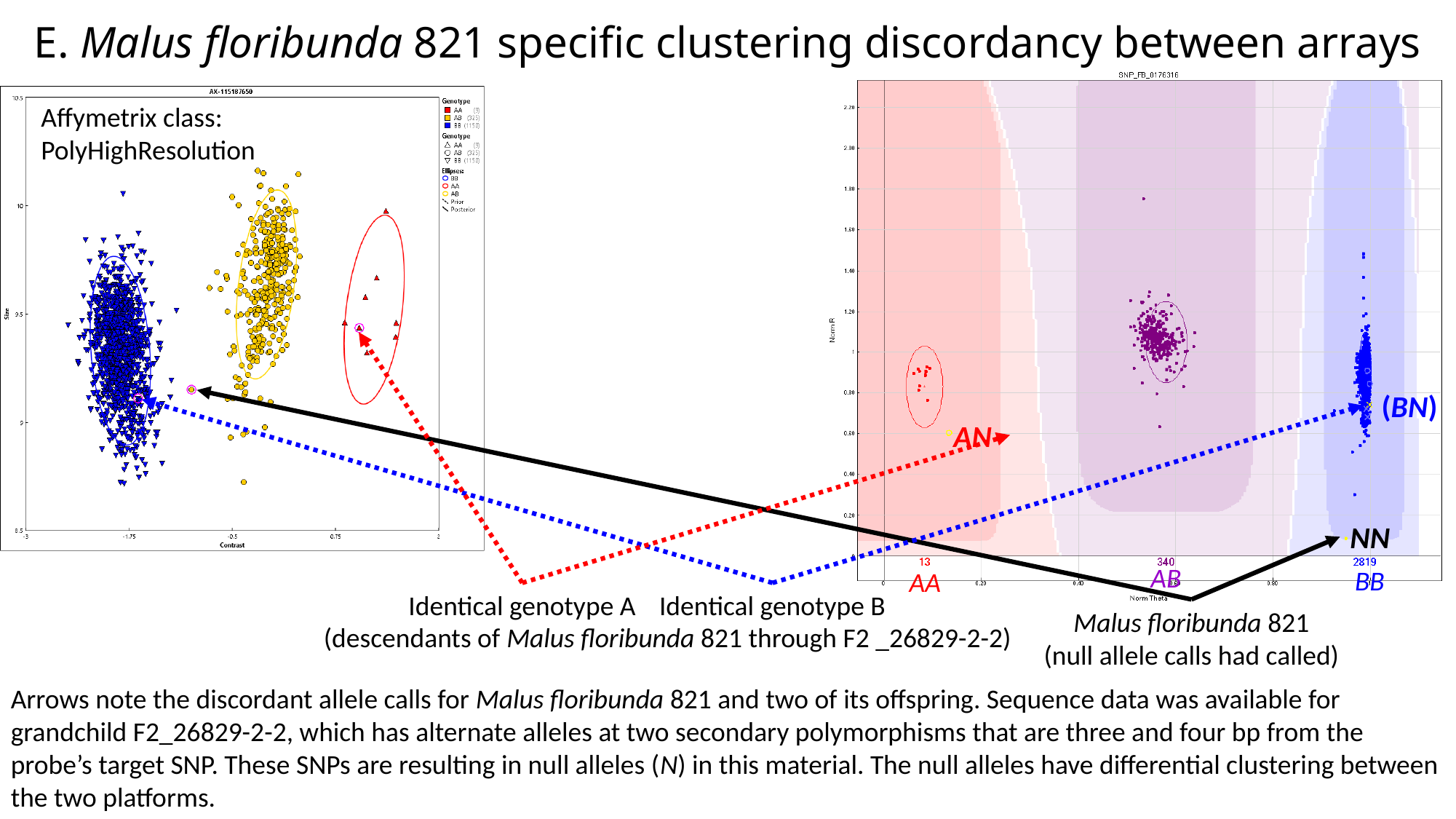

# E. Malus floribunda 821 specific clustering discordancy between arrays
Affymetrix class:
PolyHighResolution
(BN)
AN
NN
AB
BB
AA
Identical genotype A
Identical genotype B
Malus floribunda 821
(null allele calls had called)
(descendants of Malus floribunda 821 through F2 _26829-2-2)
Arrows note the discordant allele calls for Malus floribunda 821 and two of its offspring. Sequence data was available for grandchild F2_26829-2-2, which has alternate alleles at two secondary polymorphisms that are three and four bp from the probe’s target SNP. These SNPs are resulting in null alleles (N) in this material. The null alleles have differential clustering between the two platforms.

## Slide 16
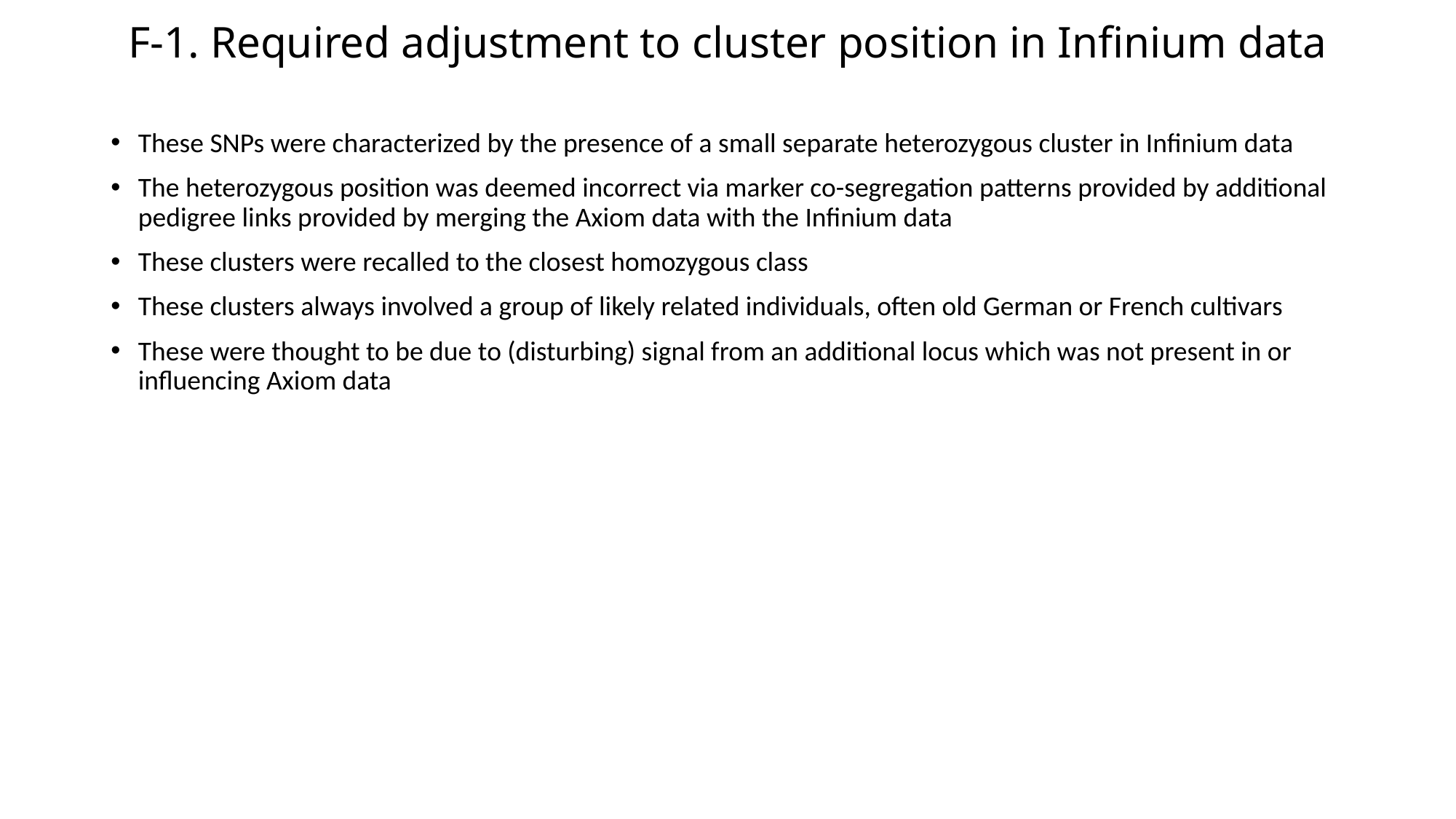

# F-1. Required adjustment to cluster position in Infinium data
These SNPs were characterized by the presence of a small separate heterozygous cluster in Infinium data
The heterozygous position was deemed incorrect via marker co-segregation patterns provided by additional pedigree links provided by merging the Axiom data with the Infinium data
These clusters were recalled to the closest homozygous class
These clusters always involved a group of likely related individuals, often old German or French cultivars
These were thought to be due to (disturbing) signal from an additional locus which was not present in or influencing Axiom data

## Slide 17
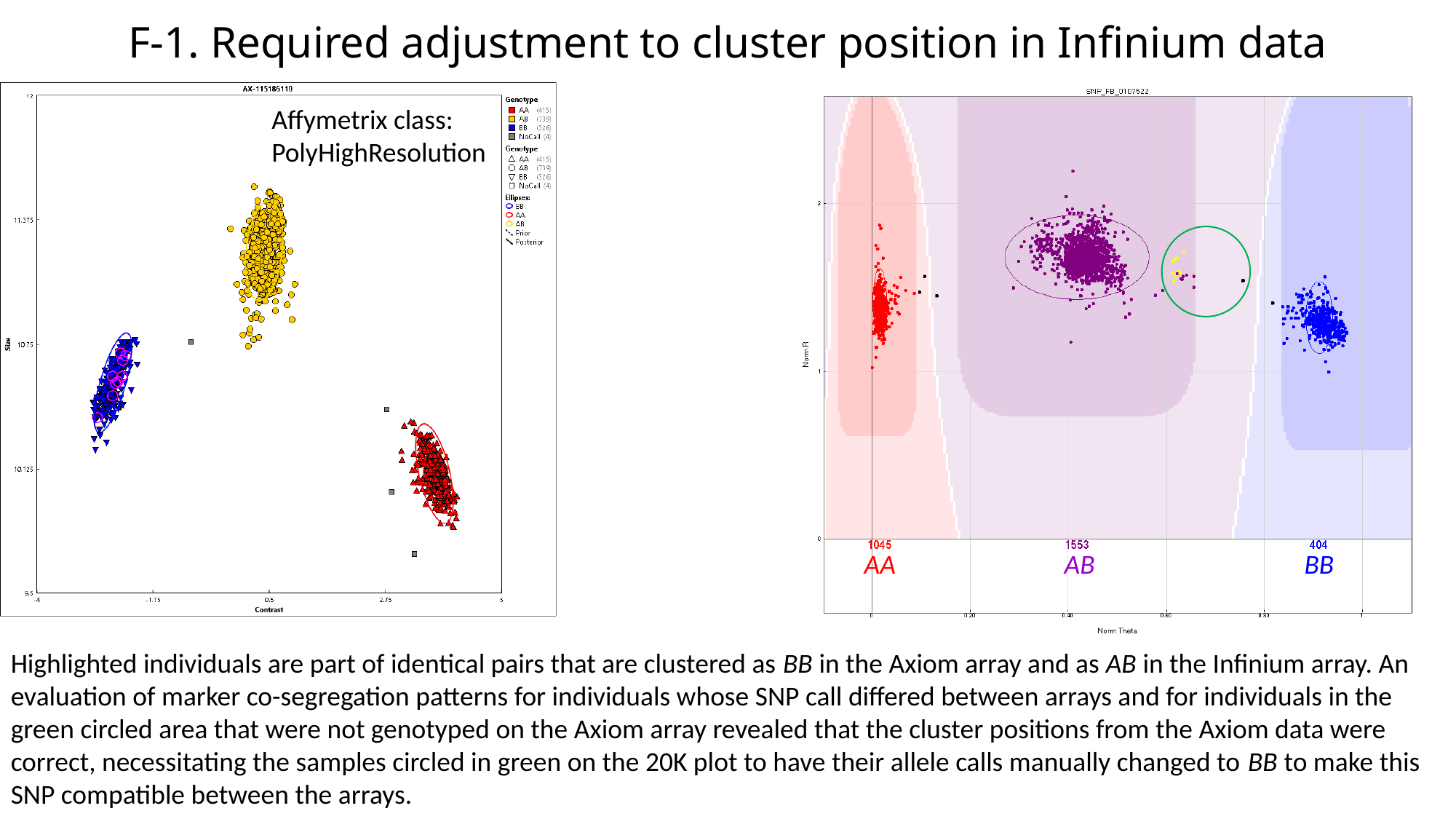

# F-1. Required adjustment to cluster position in Infinium data
Affymetrix class:
PolyHighResolution
AA
AB
BB
Highlighted individuals are part of identical pairs that are clustered as BB in the Axiom array and as AB in the Infinium array. An evaluation of marker co-segregation patterns for individuals whose SNP call differed between arrays and for individuals in the green circled area that were not genotyped on the Axiom array revealed that the cluster positions from the Axiom data were correct, necessitating the samples circled in green on the 20K plot to have their allele calls manually changed to BB to make this SNP compatible between the arrays.

## Slide 18
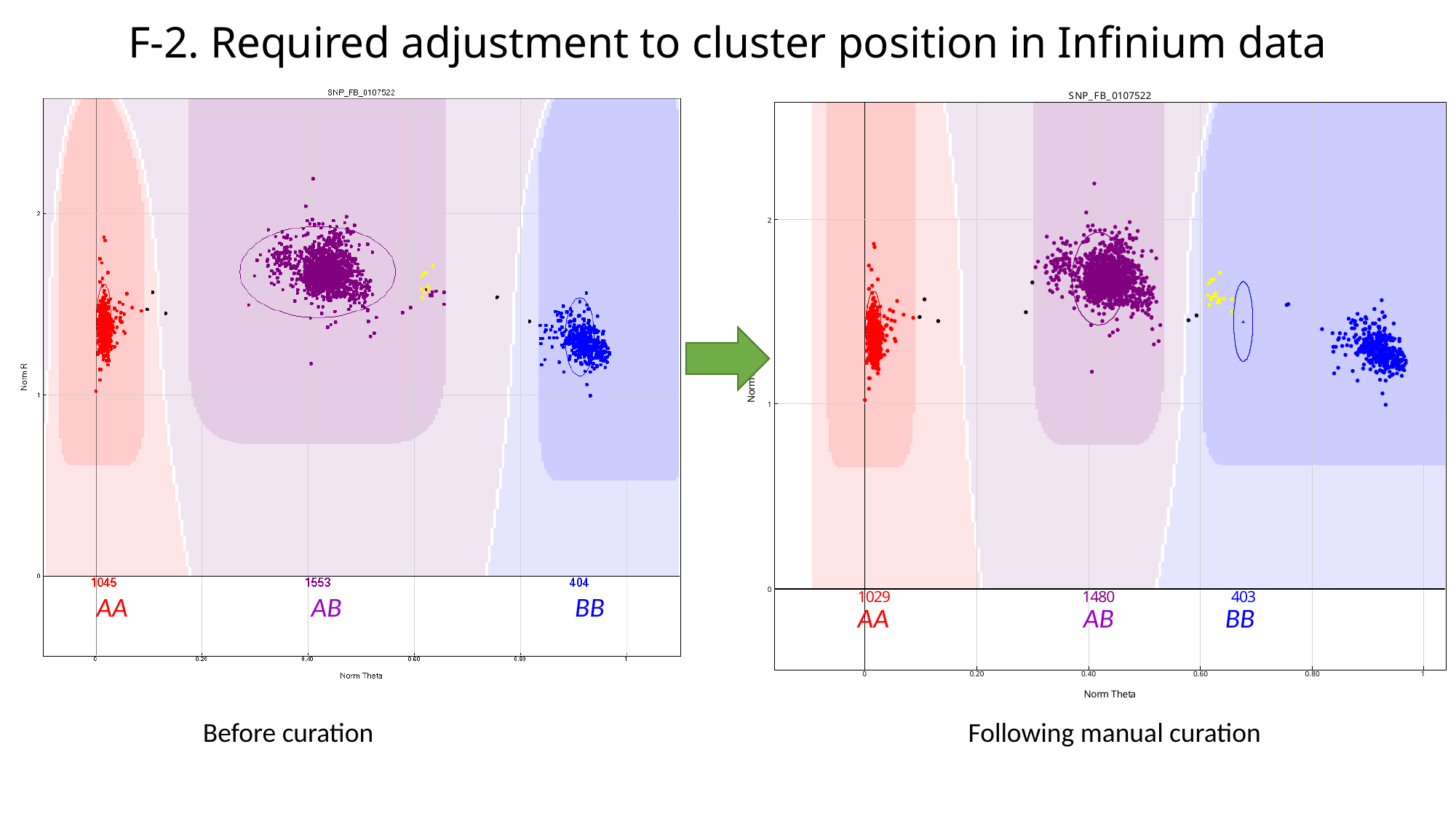

# F-2. Required adjustment to cluster position in Infinium data
AA
AB
BB
AA
AB
BB
Before curation
Following manual curation

## Slide 19
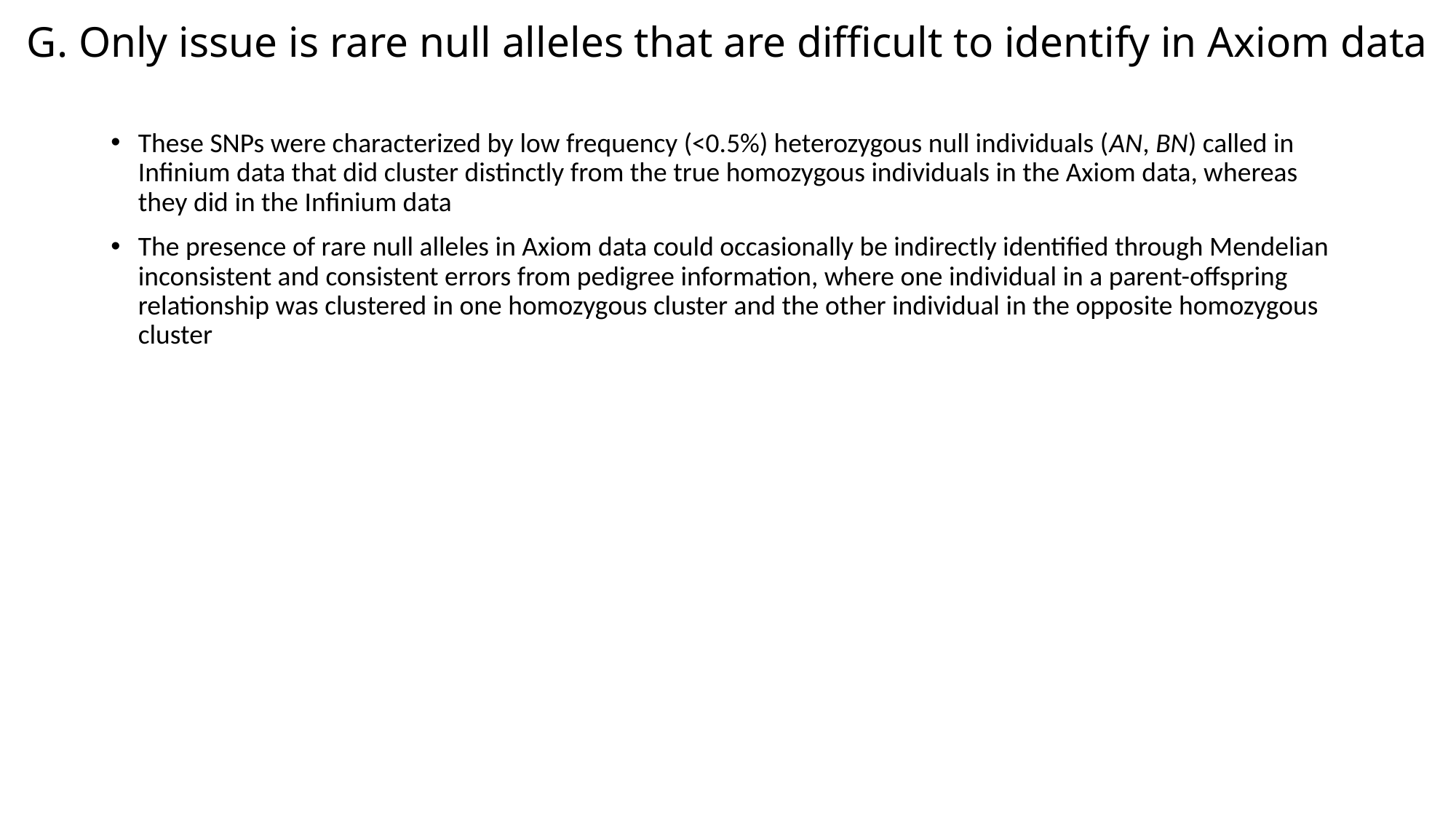

# G. Only issue is rare null alleles that are difficult to identify in Axiom data
These SNPs were characterized by low frequency (<0.5%) heterozygous null individuals (AN, BN) called in Infinium data that did cluster distinctly from the true homozygous individuals in the Axiom data, whereas they did in the Infinium data
The presence of rare null alleles in Axiom data could occasionally be indirectly identified through Mendelian inconsistent and consistent errors from pedigree information, where one individual in a parent-offspring relationship was clustered in one homozygous cluster and the other individual in the opposite homozygous cluster

## Slide 20
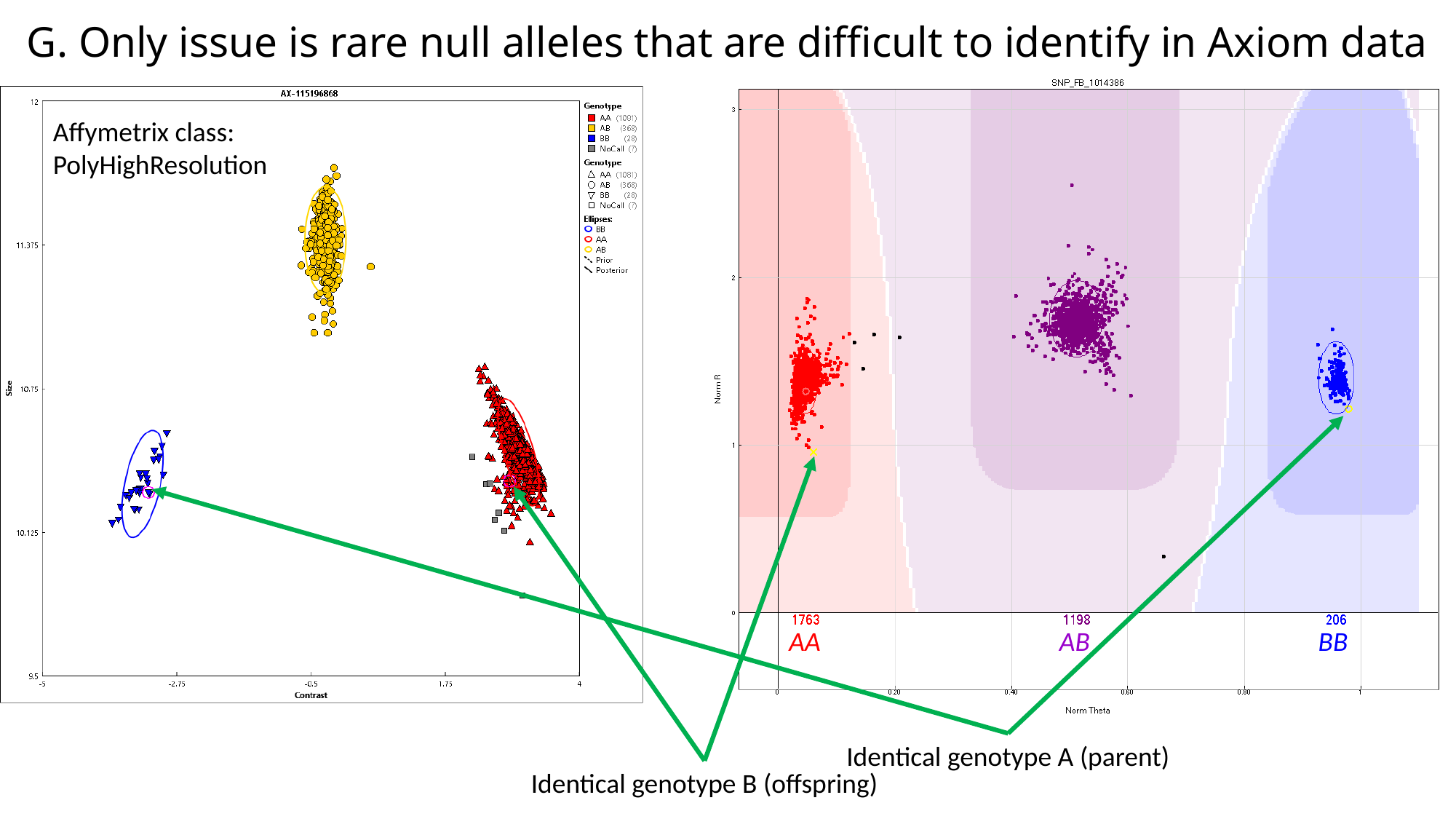

# G. Only issue is rare null alleles that are difficult to identify in Axiom data
Affymetrix class:
PolyHighResolution
AA
AB
BB
Identical genotype A (parent)
Identical genotype B (offspring)

## Slide 21
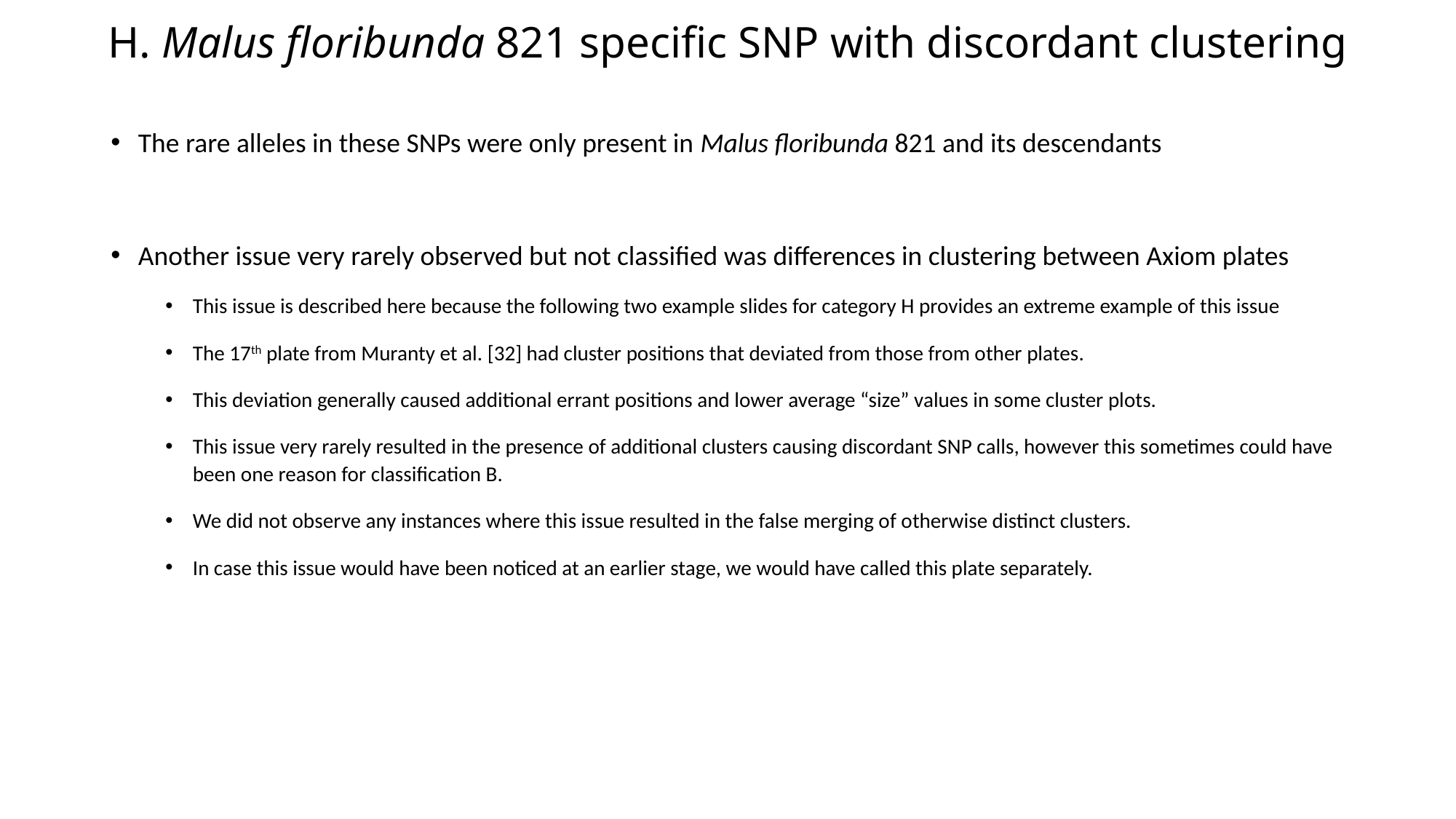

# H. Malus floribunda 821 specific SNP with discordant clustering
The rare alleles in these SNPs were only present in Malus floribunda 821 and its descendants
Another issue very rarely observed but not classified was differences in clustering between Axiom plates
This issue is described here because the following two example slides for category H provides an extreme example of this issue
The 17th plate from Muranty et al. [32] had cluster positions that deviated from those from other plates.
This deviation generally caused additional errant positions and lower average “size” values in some cluster plots.
This issue very rarely resulted in the presence of additional clusters causing discordant SNP calls, however this sometimes could have been one reason for classification B.
We did not observe any instances where this issue resulted in the false merging of otherwise distinct clusters.
In case this issue would have been noticed at an earlier stage, we would have called this plate separately.

## Slide 22
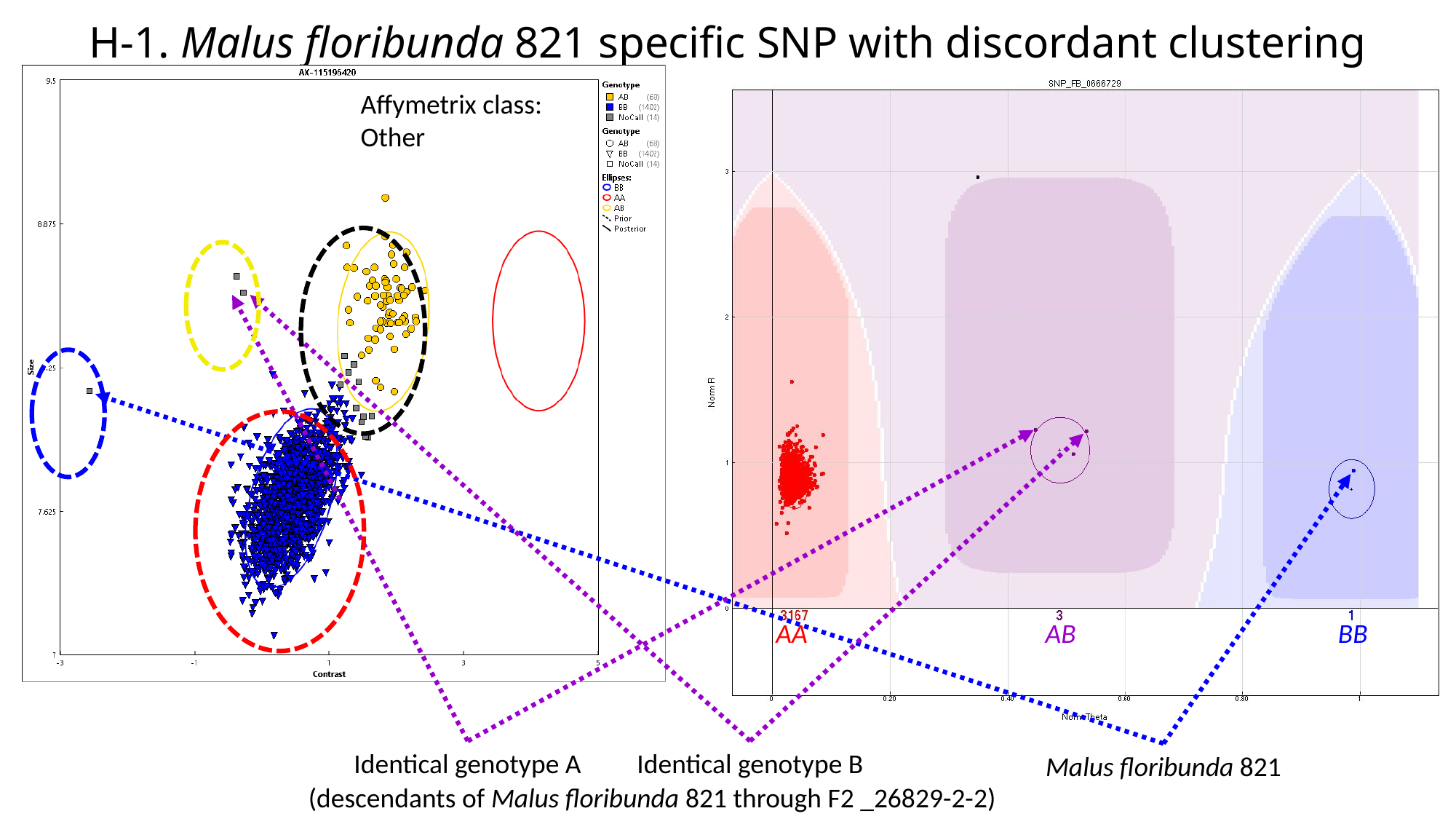

# H-1. Malus floribunda 821 specific SNP with discordant clustering
Affymetrix class:
Other
I could also include an example of
assumed null alleles from 480K relationships
AA
AB
BB
Identical genotype A
Identical genotype B
Malus floribunda 821
(descendants of Malus floribunda 821 through F2 _26829-2-2)

## Slide 23
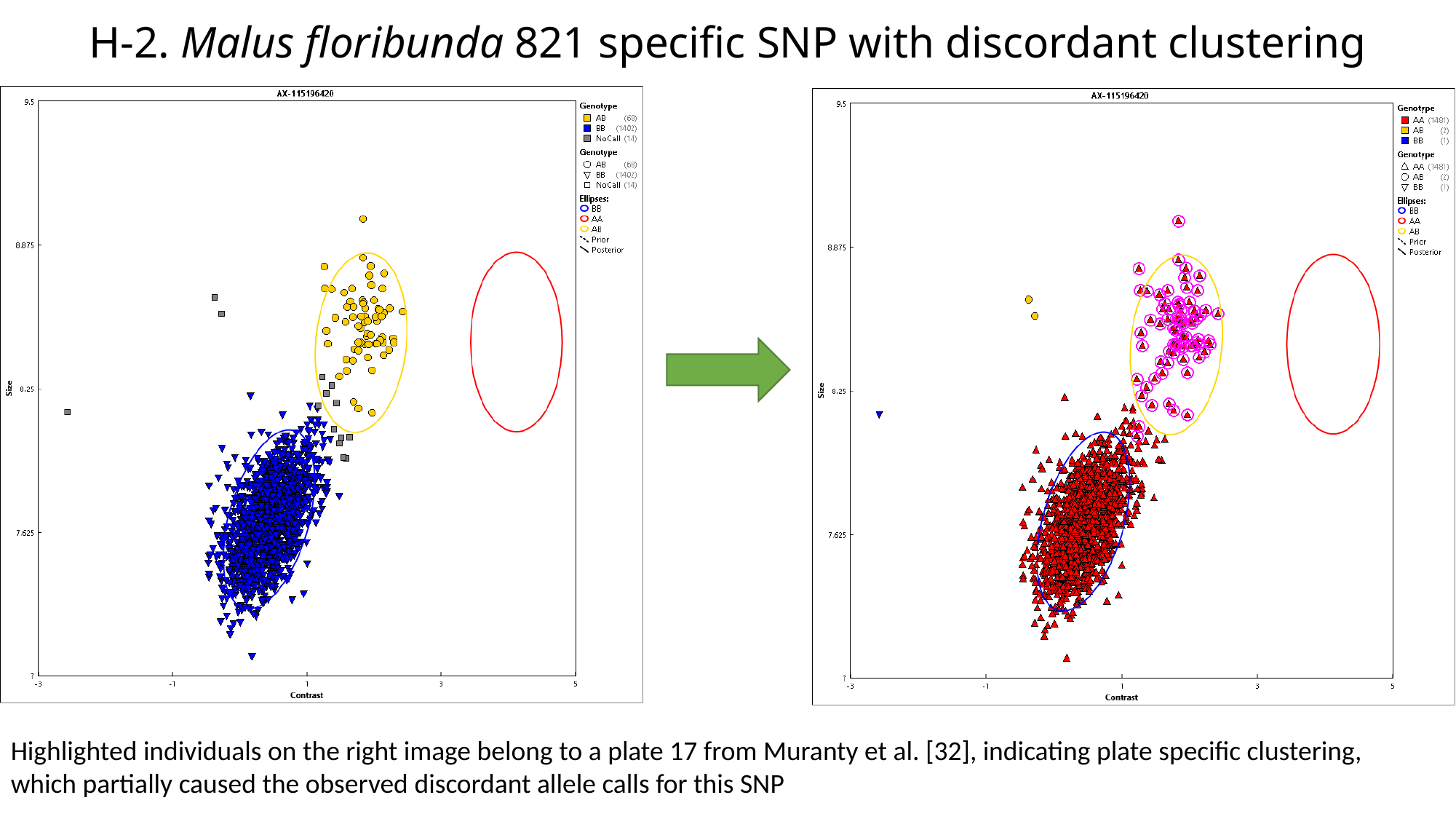

# H-2. Malus floribunda 821 specific SNP with discordant clustering
Highlighted individuals on the right image belong to a plate 17 from Muranty et al. [32], indicating plate specific clustering,
which partially caused the observed discordant allele calls for this SNP

## Slide 24
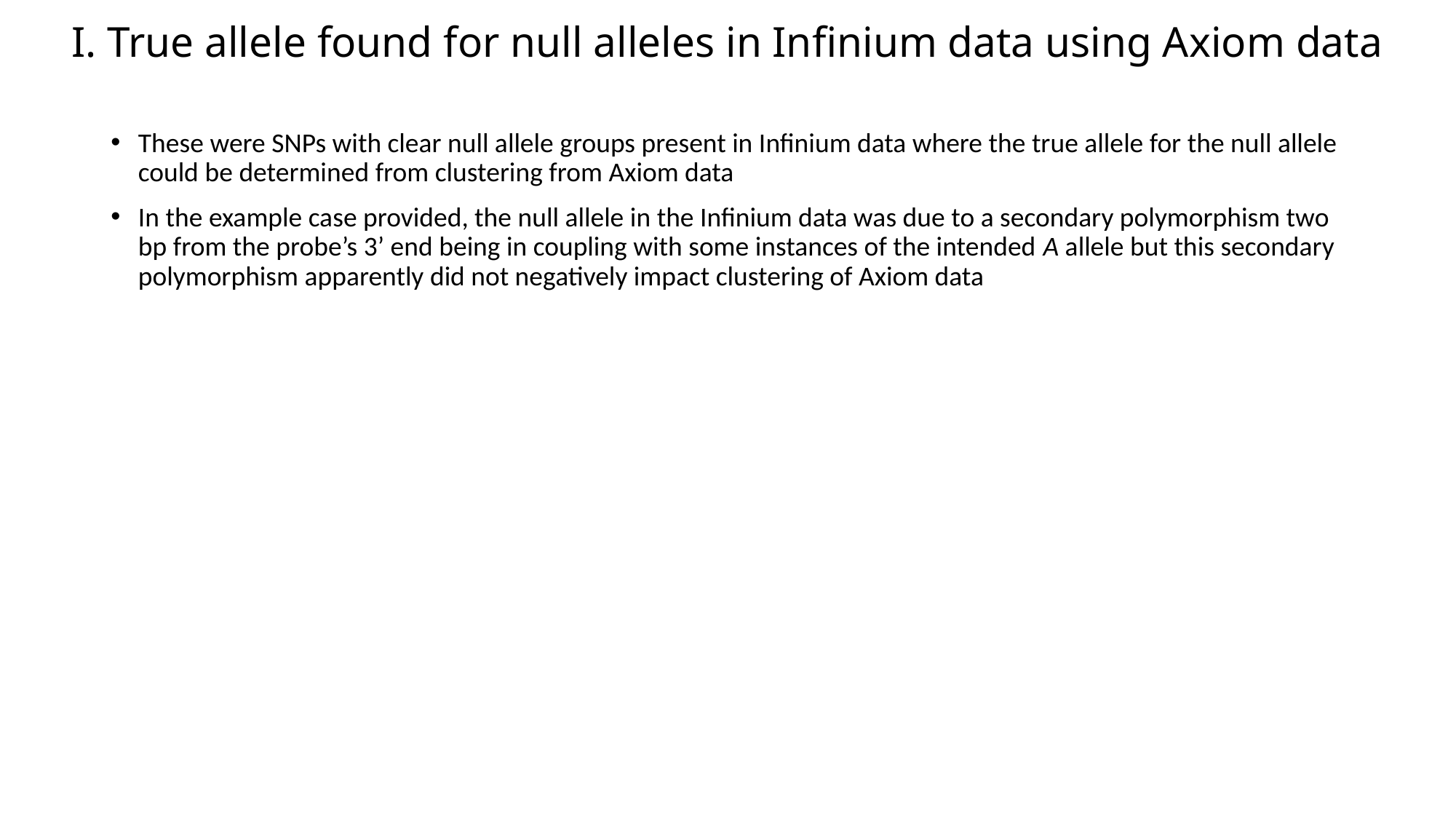

# I. True allele found for null alleles in Infinium data using Axiom data
These were SNPs with clear null allele groups present in Infinium data where the true allele for the null allele could be determined from clustering from Axiom data
In the example case provided, the null allele in the Infinium data was due to a secondary polymorphism two bp from the probe’s 3’ end being in coupling with some instances of the intended A allele but this secondary polymorphism apparently did not negatively impact clustering of Axiom data

## Slide 25
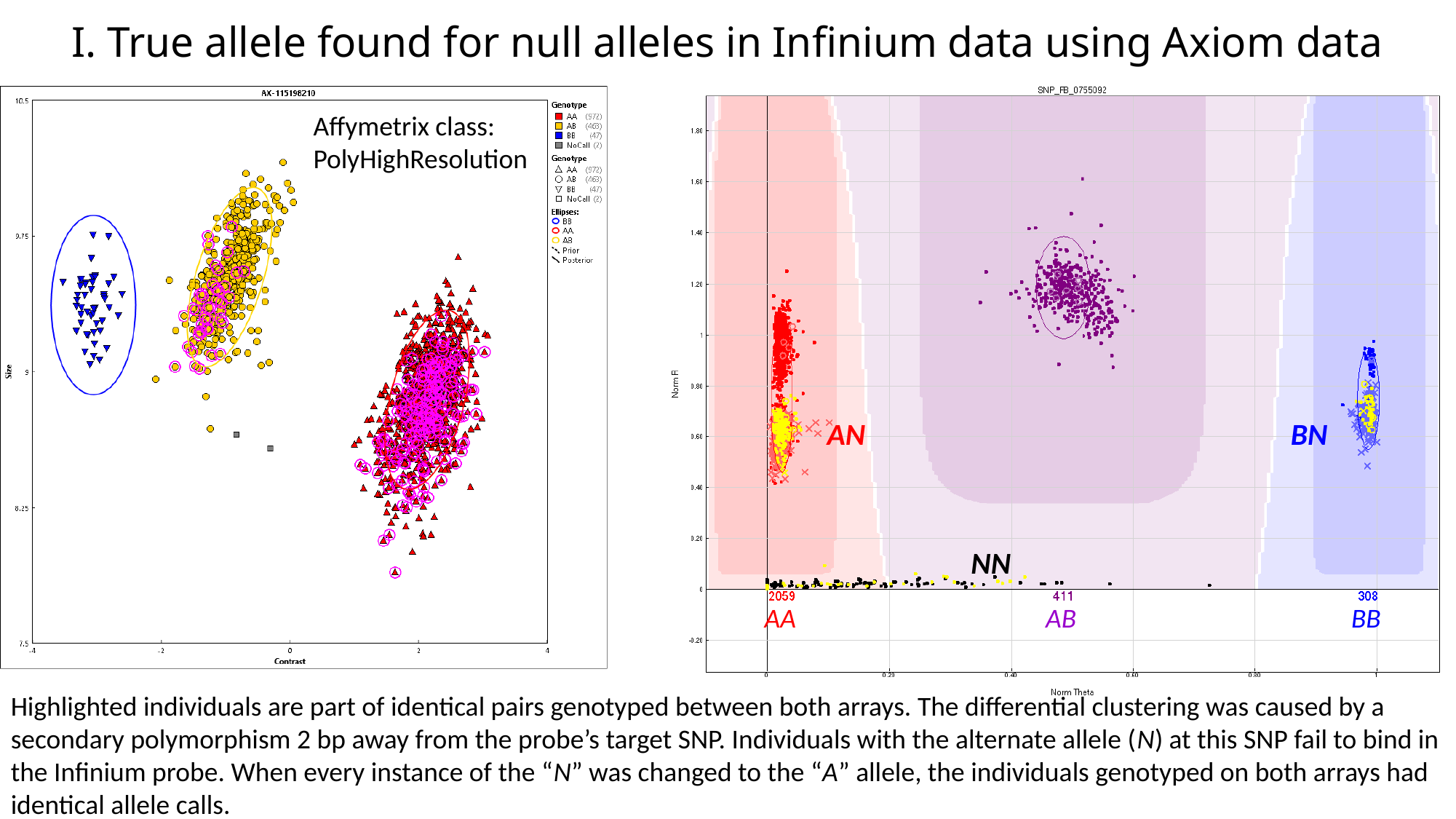

# I. True allele found for null alleles in Infinium data using Axiom data
Affymetrix class:
PolyHighResolution
AN
BN
NN
AA
AB
BB
Highlighted individuals are part of identical pairs genotyped between both arrays. The differential clustering was caused by a secondary polymorphism 2 bp away from the probe’s target SNP. Individuals with the alternate allele (N) at this SNP fail to bind in the Infinium probe. When every instance of the “N” was changed to the “A” allele, the individuals genotyped on both arrays had identical allele calls.
